# The forensic assessment of human trafficking víctims in Catalonia (Spain): characteristics and mental health status

**DOI:** 10.1192/j.eurpsy.2024.347

**Published:** 2024-08-27

**Authors:** A. Bertomeu, E. Cano, A. Mateu, A. Xifró, E. Barbería

**Affiliations:** ^1^ Institut de Medicina Legal i Ciències Forenses de Catalunya; ^2^Universitat de Barcelona, Barcelona; ^3^Universitat Rovira i Virgili, Reus, Spain

## Abstract

**Introduction:**

Systematic reviews show a high prevalence of mental distress among victims of human trafficking. In criminal proceedings in Spain, a forensic expert assessment of survivors may be ordered by the courts. Its aims are mainly, albeit not exclusively, to determine the consequences of trafficking on the physical, mental, and social health of the victims. The Institute of Legal Medicine and Forensic Sciences is the public institution providing psychiatric expert assessments in the autonomous region of Catalonia (Spain). Recently, a unit devoted to the forensic assessment of human trafficking victims has been created at the central headquarters of the Institute in Barcelona.

**Objectives:**

To describe the characteristics and the mental health status of trafficked people identified as victims in criminal proceedings.

**Methods:**

Retrospective study of case records of victims of human trafficking at the Institute of Legal Medicine and Forensic Sciences of Catalonia (2016-2023).

**Results:**

Case records of 50 survivors were identified. 38 (76%) were female; mean age was 30,5 years (SD 10,42; range 17 to 69 years). All of them were foreigners, mainly from Latin America (24; 48%). Most of them were trafficked for sexual exploitation (32; 64%). 11 (22%) were assessed immediately after their rescue (emerging cases). Some of the victims had previous mental health problems, including intellectual disability (3; 6%). Among the non-emerging cases, the most frequently recorded mental disorders at the moment of the psychiatric expert assessment were post-traumatic stress disorder (PTSD; 21; 53,8%) and anxiety (12; 30,8%). Complex PTSD was observed in 1 case.

**Conclusions:**

The majority of human trafficking survivors in the forensic setting suffer from persistent mental health problems as a consequence of their victimisation. A trauma-informed forensic psychiatric assessment is recommended.

**Disclosure of Interest:**

None Declared

